# Understanding Electronic Medical Record Adoption in the United States: Communication and Sociocultural Perspectives

**DOI:** 10.2196/ijmr.2437

**Published:** 2013-03-26

**Authors:** Priya Nambisan, Gary L Kreps, Stan Polit

**Affiliations:** ^1^George Mason UniversityDepartment of Health Administration & PolicyFairfax, VAUnited States; ^2^Center for Health and Risk CommunicationDepartment of CommunicationGeorge Mason UniversityFairfax, VAUnited States; ^3^University of PennsylvaniaUniversity of Pennsylvania Law SchoolPhiladelphia, PAUnited States

**Keywords:** electronic health records adoption, communication, systems approach

## Abstract

**Background:**

This paper adopts a communication and sociocultural perspective to analyze the factors behind the lag in electronic medical record (EMR) adoption in the United States. Much of the extant research on this topic has emphasized economic factors, particularly, lack of economic incentives, as the primary cause of the delay in EMR adoption. This prompted the Health Information Technology on Economic and Clinical Health Act that allow financial incentives through the Centers of Medicare and Medicaid Services for many health care organizations planning to adopt EMR. However, financial incentives alone have not solved the problem; many new innovations do not diffuse even when offered for free. Thus, this paper underlines the need to consider communication and sociocultural factors to develop a better understanding of the impediments of EMR adoption.

**Objective:**

The objective of this paper was to develop a holistic understanding of EMR adoption by identifying and analyzing the impact of communication and sociocultural factors that operate at 3 levels: macro (environmental), meso (organizational), and micro (individual).

**Methods:**

We use the systems approach to focus on the 3 levels (macro, meso, and micro) and developed propositions at each level drawing on the communication and sociocultural perspectives.

**Results:**

Our analysis resulted in 10 propositions that connect communication and sociocultural aspects with EMR adoption.

**Conclusions:**

This paper brings perspectives from the social sciences that have largely been missing in the extant literature of health information technology (HIT) adoption. In doing so, it implies how communication and sociocultural factors may complement (and in some instances, reinforce) the impact of economic factors on HIT adoption.

## Introduction

The slow adoption of electronic medical records (EMR) has become a critical challenge in the health care industry of the United States. [1]. Quicker adoption of EMR is necessary to streamline key processes in the health care industry, integrate activities across health care organizations, reduce overall health care costs, and improve care quality.

The US Government has made considerable efforts to improve the rate of EMR adoption [2-4]. The most recent effort was to provide financial incentives to health care organizations to implement these technologies under the Health Information Technology on Economic and Clinical Health (HITECH) Act [5]. This has led to a marginal improvement in the EMR implementation rate, but the adoption lag persists [6]. The financial incentives for adoption are available only if health care organizations agree to meet the “meaningful use” criteria set forth by the Centers of Medicare and Medicaid Services (CMS), which outlines a set of requirements (classified under stage1, stage 2, and stage 3) that would demonstrate meaningful use of the certified EMR technology. Failure to meet those criteria will lead to loss of financial incentives with detrimental effects on the successful adoption of EMR [7]. A recent study reports that many health care organizations that are getting such incentives do not plan on implementing meaningful use stage I [8]. Further, a significant number of long-term health care providers such as nursing homes, home health agencies, long-term acute care hospitals, and inpatient rehabilitation hospitals are not eligible for incentives given their lack of Medicare and Medicaid patient mix, leaving the overall EMR adoption rate “dismally low” [7]. The latest report from the Centers for Disease Control and Prevention and other studies show that another major part of the health care sector, small practice physicians, also has a very low EMR adoption rate [6,9]. Thus, despite all recent efforts, the EMR adoption lag persists.

Even among those health care organizations (HCOs) that have made efforts to implement EMR, there is a very high failure rate—studies show that up to 80% of EMR implementations fail [10,11]. Accurate estimates of implementation failures are difficult to find, as many HCOs are reluctant to report it. Approximately 19% of EMRs are uninstalled after implementation, and approximately 30% are not used to their full potential by the care staff [12]. Further, many hospitals, especially Critical Access Hospitals, were found to be lacking the technological pre-conditions required for achieving meaningful use [13]. Thus, it is clear that despite the financial and other incentives provided by the government, the adoption of EMRs remain quite problematic. A major reason for this is the lack of a clear understanding of all the factors that are likely to affect EMR adoption.

The extant research on EMR adoption suffers from a “silo” effect, typically focusing on variables drawn from a single theoretical perspective or on adoption barriers that affect a limited set of EMR’s diverse and numerous stakeholders [14]. For example, a large set of studies had drawn on Rogers’ [15] diffusion model (which focuses on individual level factors) and consequently employed a “physician as adopter” perspective to examine physician resistance to EMR [2-4,16-18]. Findings from these studies indicated several individual factors impeding EMR adoption, including concerns over computers affecting work flow, concerns about computers interfering with physician-patient interactions, limited computer literacy of physicians, and apprehension about the often unclear benefits of the new technology. At the same time, these studies seem to have ignored the existence of important organizational level factors impeding adoption such as limited return on investment, high cost of technology adoption, lack of resources, and misaligned incentive structures [2-4,19,20]. Similarly, another set of studies [11,21-23] adopted an economic perspective and institution level focus, ignoring the potential impact of non-economic and individual level factors. Most of these studies ignored the importance of environmental (or sector level) factors such as the adoption of technology and process standards in the health care industry [19]. Hence, we need a systems perspective to understand the impact of each factor at the macro or environmental level, meso or organizational level, as well as at the micro level [24].

While there is extant research on many organizational and individual factors, the studies focusing on *economic factors* received the most attention. These studies indicated that adoption is dependent on the cost effectiveness of the innovation (ie, EMRs) [3,11,21-23] and on economic incentives [25]. However, from the communication literature, we know that an innovation may not get adopted even when offered free of cost, if the adopters have inadequate information or knowledge regarding the innovation or if they do not understand the benefits of adopting the innovation [15,24,26]. Such a communication perspective (that also incorporates knowledge transfer) could shed light on the current state of EMR adoption that is lagging even after providing financial incentives [27-30]. Similarly, the sociological (or sociocultural) perspective emphasizes that innovation adoption is situated in a social (cultural) context and implies that the norms and values of the individual, the larger community of the individual, and the organization that the individual belongs to, all can influence adoption [15,31,32]. Hence, to understand the impediments of EMR adoption fully, it is necessary to incorporate complementary theoretical perspectives—particularly behavior science and sociocultural perspectives. In this paper, we apply the systems approach to analyze how communication and sociocultural factors may influence EMR adoption and offer important new insights beyond those provided by the economic perspective.

The systems approach [33] can provide an appropriate framework to develop an integrative understanding of EMR adoption—one that incorporates multiple levels of analysis. The systems approach (first proposed as the "General System Theory" by the biologist Ludwig von Bertalanffy) looks at the system as a whole instead of focusing only on individual parts. The systems perspective examines interdependent interactions among system parts as well as the interactions between the system and the environment, both in terms of system inputs and system outputs. In the context of health care, the systems approach has been successfully applied to understanding issues such as patient safety, quality of care, and health outcomes [24,34]. The systems approach is also valuable for examining the ways EMR adoption involves multiple stakeholders, multiple levels of application, and highly complex technologies (ie, multiple “parts” with complex interconnections both within and across systems).

Thus, the primary objective of this paper was to apply the systems approach to examining the communication and sociocultural issues that operate at multiple levels and shape EMR adoption. Our goal was to provide an integrative framework (developed via the systems approach) that could serve as a template for guiding future studies of EMR adoption.

## A Systems Approach to EMR Adoption

In applying the systems approach here, we draw on the nested model developed by Ferlie and Shortell [35] and Kimberly & Evanisco [36]. Ferlie and Shortell’s model classified the health care system into 4 nested levels: (1) individual (patients), (2) group level (care team), (3) organization level (health care delivery system), and (4) macro level (political and economic environment). Kimberly & Evanisco classified factors that influence hospital innovation adoption on the individual, organizational, and contextual (outside forces that influence innovation adoption) levels, and stressed the importance of examining the combined effects of adoption variables across these 3 levels instead of examining them separately. Following these studies, we focused our attention on factors at 3 levels: macro (environmental), meso (organizational), and micro (individual) levels.

## Conceptual Framework and Proposition Development

At each level (micro, meso, and macro), we examined the issues and challenges from communication and sociocultural perspectives and formulated propositions that link these factors to EMR adoption. Later, we considered how the insights derived from these propositions complement those available from the economic perspective.

### Micro Level (Individual) Factors

#### General

For a hospital, the most important customers are the physicians who bring their patients to the facility, but may not work full-time on the premises. Most physicians belong to independent physician practices, small group practices, ambulatory clinics, rehabilitation clinics, or other micro level entities that are not part of a larger health care organization. Hence, many factors at the hospital organizational level do not directly influence the decisions made by individual physicians regarding technology adoption. Micro or individual level factors have received much focus in the adoption literature, especially in the area of physician resistance, lack of computer skills, cost and return on investment of EMRs, loss of productivity caused by EMRs, and the characteristics of the technology itself (eg, [17,37]). However, there has not been much focus on key communication and cultural factors that could influence the adoption of EMRs by physicians.

#### Micro Level Communication Factors

##### Overview

Physicians are trained to be independent, authoritative, and decisive. They are often hard to reach through advertisements and promotions. Sometimes they resist innovation as a group, which makes mass communication methods ineffective. They may not be working in any health care organization or hospitals and hence organizational level methods are not applicable to many of them [38]. Some physicians run small practices where they interact with a few people in their profession and attend professional conferences once a year. They may also participate in training programs that offer continuing professional education (CPE) credits. As such, current communication methods and strategies may not effectively address this target population [39]. Adoption starts at the grassroots level and these physicians form the grassroots of the physician community [40-42].

Social contagion and social cohesion theory can be used to develop insights that apply at the micro level (eg, with physicians). Social contagion theory states that when people are in the proximity of others who have adopted a particular innovation, there will be an enhanced tendency to adopt [43]. The mere physical proximity transfers significant information regarding the innovation to the adoption laggard. Social cohesion theory implies the significance of the social interaction between the adopter and the non-adopter. According to this theory, if there is more empathetic communication between these two entities (the adopter and the non-adopter), then there is a higher chance of adoption of the innovation by the laggard. This has been shown to be quite effective in the classic adoption of tetracycline [44,45]. The autonomous nature of physicians often makes it difficult to precipitate peer-to-peer discussions about issues regarding technology adoption. Nevertheless, social networks, virtual communities, and social media can be used in the diffusion of innovation among this group [40].

The establishment of 62 Regional Extension Offices through the 2009 HITECH Act was a significant step forward in employing communicative approach to promoting adoption of EMRs. The objective of this program was to reframe the national issue of technology adoption, and facilitate dialogue on a regional level, thus encouraging discussion of unique local factors influencing EMR adoption. However, we are not sure whether these extension offices are effectively communicating EMR information availability, as many regional websites do not even provide the required information for meeting meaningful use criteria (eg, Alabama regional extension center opened in 2010 does not contain this information).

The regional extension offices, if used effectively, could have multilayered benefits for diffusing relevant information about the need for EMRs. These offices can place physicians in the role of “leaders”, allowing them to become what Rogers [15] referred to as “change agents”, or individuals who have the ability to influence the decisions of others. Even though the federal government is facilitating and funding this program, having physicians disseminate technology adoption messages and facilitate discussions of the benefits and barriers to EMR will create a more authentic and convincing argument. Because physicians can share practical and implementation concerns among themselves, physicians are the key players to stir initial interest regarding technology. The federal government can then serve their role in supplying tools and incentives to further facilitate the technology promotion and implementation process. This process is referred to as the social cognitive method, using a socially mediated pathway to connect audiences through social networks that provide continued reinforcements for desired change [46]. Rogers [15] also advocated for this diffusion strategy because familiar interpersonal sources are more effective in inspiring individuals to accept new ideas than when discussions from more distant sources. The use of identifiable change agents could also promote further diffusion of EMR adoption by encouraging continuous recruitment of new opinion leaders to carry EMR messages from each physician-physician communication cycle.

##### Proposition 1

Communication tools such as social media (which is the fastest tool for the social contagion and social cohesion methods) will be positively related to the adoption and diffusion of EMRs among independent physicians in small practice settings.

##### Proposition 2

Implementation of communication mechanisms that function at the grassroots level and target independent physicians to promote and facilitate EMR use will be positively related to the adoption and sustained use of EMR by small practice physicians.

#### Micro Level Cultural Factors

##### Overview

Despite a considerable number of studies addressing other factors associated with EMR adoption, research on the topic has often overlooked the readiness of physicians to serve as the key implementers of EMR systems [21]. For physicians, there currently exists a culture of apprehension and distrust that permeates the adoption of EMR technology [47]. Shachak and Reis [48] elaborated that these feelings make it highly pertinent to understand how the cognitive elements of implementation shape perceptions of barriers.

Some individual cultural impediments to adoption stem from physician perceptions that these systems may challenge their authority as autonomous decision makers in the delivery of care. A key part of the physician psyche is how they cherish and protect their role as the expert in the care provider scenario. Unfortunately, a lack of understanding regarding technology, specifically how they should integrate EMR systems into their work, often leads physicians to view themselves as novices in this area. The juxtaposition between concurrent roles of “expert” and “novice” creates a high degree of cognitive dissonance for physicians [49]. One proposed solution is to place physicians at the forefront of efforts to address the cognitive impediments to technology adoption [50]. A benefit of this approach is that physicians begin to develop a sense of psychological ownership over the development and use of EMRs [51]. Ludwick and Doucette [47] advocated for this kind of approach by explaining how the most effective changes in the health care system occur when physicians are at the helm. Thus, framing physicians as leaders in adoption efforts allow them to become the principle force influencing the future of medical practice.

Cultural issues involving small practice physicians follow closely with the needs of independence and autonomy. While many physicians are attracted to the autonomy and independence small practices provide, they are also wary of the challenges of sustainability, with many small practices across the country going bankrupt or getting bought by large health care organizations [52,53]. Issues such as rising business expenses and administrative costs are cited for the demise of many small practices. However, many of these practices have not changed much in the past several decades in the way they practice or conduct business. Competition from new models of care such as walk-in clinics and practices run by large health care centers require that small physician owned practices keep up with the changing health care environment as well as with changing consumer needs. Consumers are likely to increasingly seek care at walk-in clinics and urgent care centers attracted by their convenient hours and quick service. Many walk in clinics and urgent care centers tout that their patients are using them for primary care and many of them provide continuity of care by relying on technologies such as EMRs. The lack of entrepreneurship skills, lack of customer orientation, and lack of understanding that technologies such as EMR are soon going to be a necessary infrastructure rather than a luxury [54], could be some of the reasons why small practice physicians are lagging behind in EMR adoption. Many experts also believe that small medical care practices that survive would need to stay connected or affiliated with other small practices through mechanisms such as shared EMRs [53].

One of the key issues that need to be addressed is the need for change in the ‘culture of small medical practice’ businesses. Many small practice owners need entrepreneurship skills and training on how to conduct business in the Internet era. Cultural change in customer orientation, entrepreneurship orientation, and perceptions regarding new technologies could be some of the factors that could lead to higher adoption of EMRs among this group.

##### Proposition 3

The level of physician involvement at the grassroots level in the initial adoption process will be positively related to the overall adoption and sustained use of EMRs by physicians. The decision-making power of physicians during these initial adoption stages is crucial for the success of EMR adoption.

##### Proposition 4

Cultural change in customer orientation, entrepreneur orientation, and change in perception of new technologies will be positively related to the adoption of EMRs among small practice physicians.

### Meso Level (Organizational) Factors

#### General

Organizational researchers have studied a multitude of factors that could influence the adoption of new technological innovations in organizations [31,32,36,55]. These factors range from characteristics of the organization itself to the composition of its employees to organizational leadership and resource availability.

#### Organizational Level Communication Factors

##### Overview

The adoption of complex technologies such as EMR calls for effective communication among adopters and the potential for transferring experiential knowledge and learning. Such a communication and knowledge transfer perspective of technology adoption also ties in well with the notion that health care organizations need to increasingly become learning organizations to enforce radical changes and bring about transformation in services, practices, and processes [56]. There are several barriers to establishing a learning culture in health care organizations [57,58], ranging from the complex hierarchical work structure to physician resistance towards learning and sharing knowledge.

An important factor that affects learning is the mode of communication in the health care organization. Much of the information flow within a hospital involves health care workers communicating directly with one another [59]. In fact, face-to-face communications constitute half of such communications, while communication through electronic devices (pagers, phones, etc) accounts for the other half [59,60]. With the increasing number of staff and hospital workers, this type of communication (face-to-face or phone) has been found to be highly interruptive and is a leading cause of errors. Coiera and Tombs [60] observed that communication among employees in a hospital environment often leads to interruption-driven work contexts, where miscommunication or ineffective communication is the norm. Thus, in this kind of environment, getting physicians and other staff to communicate with one another and engage in knowledge sharing becomes challenging and potentially makes EMR adoption very difficult.

There is a critical need for health care organizations to implement good communication policies that are engaging and productive rather than disruptive [59]. The provision of a communication infrastructure that utilizes new communication technologies may enable health care workers to not only communicate important task-related messages, but also take part in other productive conversations. Evidence indicates that online communities and communities of practice where physicians can share information through online forums have the potential to address many of the deeply rooted cultural factors that inhibit the development of a learning culture in health care organizations [61-63]. Such forums allow adopters of new technology to not only share their experiences related to the new technology, but also describe their own innovations or reinventions.

It is well established that adopters of new innovations often learn by using the innovation [64,65] or reinvent the technology to adapt it to their own context [66,67]. The ability to share such user innovations and experiences are invaluable during the adoption of new technologies such as EMR. There are some online forums such as the Paperless Practice Groups that provide user support for EMR adoption issues, but this could be supplemented by online support groups within the organization where users can share issues and problems while using the new technology at their specific institution to help each other. Here we suggest that the availability of such diverse communication forums can enhance learning related to EMR deployment and lead to faster EMR adoption.

##### Proposition 5

Facilitating a learning environment by offering diverse knowledge sharing facilities such as online forums will be positively related to EMR adoption at the organizational level.

#### Organizational Level Cultural Factors

##### Overview

An organizational culture that fosters leadership and support is a critical factor when it comes to technology adoption. For example, Rogers’ authority innovation-decision model [15] shows that leaders use their authority to enforce change. Peter Senge’s [68] concept of leadership in a learning organization also illustrates how leaders are supposed to steward and teach members, thereby driving adoption. Further, in the innovation adoption literature, characteristics of key organizational actors have been found to be critical in influencing the innovative behavior of people within the organization and thereby their willingness to engage in adoption processes [69-71].

In the case of EMR, “adoption by fiat” has been found to be quite effective. The classic example was the Veterans Administration (VA) system, where the top leadership decided to adopt and implement EMRs and the physicians and other staff members were required to comply as system employees [72]. Unfortunately, such a scenario is unlikely to exist in most EMR adoption contexts since in most health care organizations, physicians (who are the users/adopters) are partners or stakeholders, instead of employees.

Prior research [73] indicated that a key factor that could facilitate adoption in such contexts is the extent of *user involvement* in the adoption process. Several examples clearly show that involving physicians and other administrators during the EMR adoption decision-making process can go far in enhancing their motivation to adopt [74]. Practices such as listening to stakeholder concerns, inviting physicians, and other staff to make adoption recommendations, and including having users as implementation team members, have all been found to enhance the adoption rate [75]. Palacio et al [75] suggested that a forum for multidisciplinary information planning committees could encourage such user-driven discussions. By bringing together various types of health care stakeholders, it becomes possible to uncover a wide range of experiences regarding the institutional integration of technology into care delivery.

Another critical adoption factor is at the level of organizational commitment and support. In the context of EMR, organizations could invest in support facilities such as help desks and online user communities that help organization members address implementation concerns. Additionally, the level of technology training offered by management is another important factor cited for successful adoption of EMRs. For example, the VISTA system at the VA, is considered to be one of the most successful EMR implementations, touts its training program as a critical success factor in implementation [72]. Similarly, organizational leaders need to develop and communicate a shared vision and understanding of EMR adoption and use within the organization—a vision that connects EMRs with the organizational (or business) mission and objectives. Such a shared vision could bring congruence to the activities associated with EMR adoption across different functions or departments within the organization and enable faster and smoother adoption.

##### Proposition 6

Development of a participatory work environment that promotes organizational members’ active involvement in the EMR adoption and implementation process and decision-making will be positively related to the adoption and sustained use of EMRs.

##### Proposition 7

The commitment and support of organizational leaders (through deployment of explicit support mechanisms and the communication of a shared vision for EMR adoption) will be positively related to the adoption and sustained use of EMRs.

### Macro Level (Environmental) Factors

#### General

In the area of EMR adoption, the key macro level entity is the federal government which influences EMR adoption through reimbursement practices of Medicare/Medicaid and direct funding of EMR implementation.

#### Macro Level Communication Factors

##### Overview

Many new technologies and products experience an initial spurt of adoption (eg, products such as the iPod, iPhone, etc). The primary reason for such a high rate of early adoption is advertisements in mass media such as TV and magazines. This applies to EMRs as well. Many new technologies often do not catch on due to a lack of promotional efforts. For example, physician portals were developed purely out of demand from physicians who wanted to access patient records remotely. Many health care organizations developed these systems, but unfortunately, did not promote or market them [76], so the potential of this technology was never fully understood by physicians. In essence, even the cool products will not sell if there is inadequate marketing and promotional efforts emphasizing the products’ attributes.

Two factors assume importance here: the *content* of the communication and the *target* of the communication efforts [77]. The content of the communication should be able to address the complex changes and upheavals faced by health care providers, which is leading to the delay in their EMR adoption. There are a lot of new changes being implemented in the area of health care by programs such as Accountable Care Organizations (ACOs) and Patient Centered Medical Homes (PCMH) in addition to the impending changes brought down by the Affordable Care Act. Such complex changes and uncertainties could put physicians under a lot of stress and strategic communication is critical to provide clarifications. For example, one effective communication strategy would be to convey to physicians it is critical to adopt an EMR—the new models such as ACOs and PCMH depend on physician practices and hospitals that have already implemented EMRs. Hence, strategic communication efforts at the macro level should focus on both promoting EMRs (ie, its benefits and payoffs) as well as addressing the potential issues and complexities of EMR adoption.

Second, is to understand the target of the communication efforts. It is important to understand that there are multiple types of stakeholders who can influence EMR adoption by health care providers. Currently, promotional efforts primarily target physicians through medical journals and medical conferences, although, it is mostly done by the vendors who want to sell their EMR products. However, marketing and advertising efforts need not be just physician-focused. These strategies could also target additional stakeholders of care delivery. For example, direct consumer marketing has been long adopted by pharmaceutical companies and has been found to be a very effective method in not only increasing the awareness of a particular drug, but also in stirring demand for the drug and eventual sales [78]. Improving awareness among consumers about the quality difference of care by providers who have adopted EMR versus those who have not adopted EMR could be one way to increase the adoption rate among providers. The potential for implementing EMR systems that can provide relevant health information to patients is likely to be very attractive to consumers [79]. The growth of mobile health products and mHealth applications for smart phones has provided new gateways for communication between physicians and patients, which, through telemedicine, will definitely necessitate increased use of EMRs. While patient markets for mHealth apps have been aggressively marketed, the use of EMRs for hospitals has not been promoted similarly. In short, the implications of adopting EMRs go beyond one set of stakeholders and involve a diverse set of stakeholders. Therefore, mass communication campaigns that target these different stakeholders, and in some cases when targeted together, rather than separately, are likely to enhance EMR adoption rates.

##### Proposition 8

Effective communication at the macro level that focuses on both the benefits of EMRs as well as the likely challenges and complexities of EMR adoption will be positively related to the adoption of EMRs.

##### Proposition 9

Mass communication strategies at the macro level that targets not only the direct users (physicians and providers), but also other stakeholders or beneficiaries of EMR systems (including insurance companies as well as indirect users such as patients and pharmacists) will be positively related to the adoption of EMRs.

#### Macro-Level Cultural Factors

##### Overview

Currently, the macro culture in the health care industry related to EMR adoption can be described as very negative, focused on blaming individuals and institutions attributed with preventing the promotion and adoption of EMR systems. For example, Bleich and Slack [80] explained how marketing-based approaches to change physician behaviors and attitudes regarding the use of EMR technology have proven ineffective because they tend to frame physicians themselves as one of the main impediments to adoption efforts. The existence of this culture of negativity is quite evident, based on the number of articles pertaining to physician resistance and that physicians themselves are a central barrier to adoption efforts [2,17,21,80-82]. Conversely, physicians also fuel this culture by blaming insurance companies for advocating EMRs because they are the institutions most likely to reap the financial benefits of technology adoption, at least initially [19,47]. As a result of this blame shifting, altering the current dynamics of the situation requires reframing of the relationships between all stakeholders involved in the adoption process. These stakeholders include, but are not limited to, physicians, care providers, health care organizations, and government institutions that have vested interests in the development and spread of EMRs. In addition, there has been a lot more focus on implementation failures than success stories and this adds to the negative perceptions regarding EMR.

Counteracting this negative culture requires an understanding of the institutional and product related goals that perpetuate hostility towards all aspects of EMR adoption. Recognizing physicians as the end users of EMRs pushes policy makers to assure that supply-side institutions are developing products that adequately function within highly regulated and complicated medical environments. For example, O’Malley et al [83] explained that when physicians are perceived as the central barrier to EMR adoption, it only exacerbates the gap between physicians’ experiences with EMRs and policy makers’ expectations. Ludwick and Doucette [47] elaborated that not acknowledging these gaps results in supporters of EMR, possibly promoting dysfunctional systems. EMR advocates who promote technology that is misaligned with physicians’ expectations and needs, which may precipitate a vicious cycle in which ineffective systems become the gold-standard upon which all systems are associated and compared [47].

A critical challenge that physicians face when attempting to adopt EMR systems is the unwillingness of product developers and manufacturers to match their products to the individualized needs of physicians and medical groups [47]. Perceived attributes of any new innovation can influence the rate of adoption [15]. Not only do physicians struggle to find EMR systems that match their specific needs, but these systems can also be ineffective in delivering one of the most widely touted benefits, increased physician coordination. Jha et al [84] articulated how the plethora of EMR products offered to physicians often lead to use of incompatible systems between different care providers. Due to a lack of congruency between proposed EMR goals and functionality, physicians are concerned that the switch to an electronic product may create problems associated with patient privacy, physician-patient power relationships, and quality of care delivery [47,85].

Vendors should focus on not just advertising the potential benefits of EMR adoption but also ensuring that the innovation (ie, EMRs) is compatible with the broader cultural setting (ie, physician practice setting) in which it will be deployed. For example, efforts to enhance the overall compatibility of EMRs with the macro culture would likely enhance the adoption rate. Similarly vendor efforts to enhance the observability and the demonstrability of EMR technology (how will it work and what will be the potential outcomes) will likely reduce the cultural resistance to EMR adoption that is largely fueled by ignorance and suspicion. It has been found that physicians do adopt medical technologies like diagnostic tools (where the technology has immediate effects on their job outcomes) and other consumer technologies in their personal life but not EMRs, which are perceived as highly complicated, costly, and cumbersome [85]. Another source of negativity stems from stories of implementation mishaps and well-publicized implementation failures in the health care industry [10,86]. These implementation disasters are highly avoidable as their root causes are typically due to vendors’ poor understanding of the health care environment, lack of user involvement in the implementation process, and severe lack of user training that should be provided by the vendors.

##### Proposition 10

Vendor efforts to understand and align EMR technology vis-à-vis the cultural factors associated with technology adoption in the health care field as well as the work context of health care professionals will be positively related to the adoption of EMRs.

## Discussion and Implications

Understanding EMR adoption from the communication and sociocultural perspectives is very important, as there is a limit to enhancing adoption through economic or financial incentives alone. There are implications for researchers, practitioners as well as for policy makers. From a research perspective, it implies the need to further explore and investigate the communication and sociocultural factors that are relevant to EMR adoption.

The nature of EMR implementation is inherently complicated. As this study indicates, the impediments to these adoption efforts involve not only a diverse set of actors but also a complex set of interactions between these stakeholders across a variety of levels. Navigating through these barriers require advocates of technology adoption to acknowledge that these challenges cannot be isolated to any one set of variables. There needs to be more studies conducted about how we can address the myriad multi-level communication challenges and sociocultural challenges within the health care industry. While proponents of financial incentives are quick to note that there has been an increase in the rate of adoption after the implementation of the HITECH Act, it does not mean that the early adopters are going to complete the EMR implementation process, as many have raised doubts whether they will be able to meet the meaningful use criteria. However, there definitely is higher momentum than before in EMR adoption, partly due to the financial incentives and partly due to the influx of younger and more tech savvy care providers. This indicates that more research in understanding the range of key adoption and implementation factors would play a critical role in promoting or helping the adoption momentum set off by the financial incentives.

For practitioners, it is important to understand that all the issues with health information technology (HIT) adoption cannot be addressed with financial incentives alone and that it is critical to take a holistic perspective and address issues not only at the organizational level, but also at the macro and individual levels, and devise appropriate incentives at the different levels. Many organizations spend a lot of unnecessary money on EMRs due to a lack of information regarding the kind of information systems they require, lack of understanding of the needs and requirements of different types and levels of users, lack of promotional efforts after implementation, or a lack of understanding of the sociocultural aspects of users at different levels. The communication gap within and between various actors and stakeholders creates an even more complex situation, where solutions at one level or for one set of actors is rejected by another set. Hence, it is critical at this time to invest in understanding the communication/knowledge needs as well as the sociocultural factors that are relevant to technology adoption.

For policy makers, it is important to understand that while financial incentives may produce some positive results, without addressing the need for broader promotional and educational efforts, such advances in HIT, adoption may not be sustainable. At the macro level, there is some understanding of these factors and hence the provision of health information exchanges (HIEs). However, many of these HIEs are not promoted well as some do not have websites or any information that they are supposed to provide and there has been minimal formal evaluation of the effectiveness of these HIEs. HIEs will not be effective if physicians and other users do not know about the existence of these exchanges and the purpose of their existence.

Financially-oriented issues are not just found in the “carrot” of incentive-based efforts, but also in the affiliated “stick” of punishments for not meeting EMR adoption standards. A goal of federally-sponsored EMR incentives is to boost US physician adoption rates to 90% by 2020 [9,87]. To ensure that physicians are willing to utilize these incentives, the federal government will begin levying penalties on noncompliant physicians starting in 2015. These penalties will come in the form of a progressive fine starting at one percent of a physician’s Medicare receipts and increase an additional one percent each year [88]. A noted problem with this kind of approach to EMR implementation is that, as research indicates, when a hardline approach to changing physicians’ behaviors is implemented, the reaction is usually emboldened resistance [51].

Clearly, there needs to be more research on how to address the proverbial “what is in it for me” question. Instead of focusing on the cash value of adoption, there needs to be more focus on benefits other than those that are purely economic in nature [85]. Non-economic returns have driven the success of many consumer products that even physicians are attracted to and use in their daily lives [85]. Similar benefits are evident in some of the EMR technologies too, for example, in the adoption of Picture Archiving Communication Systems (PACS). Radiologists can see medical imaging pictures digitally, enlarge them on the computer screen and make more accurate diagnoses, and above all, they can do this from their own home. As a consequence of the convenience and technical advantages brought by this new technology, PACS has a high adoption rate. The example of the success of PACS adoption illustrates how vendors can promote health information technology products that deliver specific broad benefits (eg, improve quality of care, reduce errors, enhance satisfaction, reduce stress, or enhance subjective well-being) and also provide good financial investments. Mandl and Kohane [85] question the need for promotion of EMR systems to be overly complicated and call for promotion of applications similar to consumer IT products that physicians use in their daily lives.

In conclusion, financial incentives may have helped with getting the momentum started for EMR adoption to some extent, but there is a limit to the influence of such incentives. EMRs implemented without complying with meaningful use criteria will not lead to full realization of the potential of EMRs for health care practices and is not going to fully benefit patients in terms of transparency and access to records. Further, by focusing on culture and communication perspectives, we understand that monetary incentives may play only a limited role in the larger scheme of EMR adoption. Without integrating a broad range of communication and cultural factors into the promotion of EMR adoption (eg, administrative, marketing, and lifestyle benefits), it might be over ambitious to expect high results with the current financial incentives offered by the Federal government.

## Abbreviations

ACO: Accountable Care Organization
CMS: Centers for Medicare and Medicaid Services
CPE: continuing professional education
EMR: electronic medical record
HCO: health care organizations
HIE: health information exchange
HIT: health information technology
HITECH: Health Information Technology on Economic and Clinical Health
PACS: Picture Archiving Communication Systems
PCMH: patient centered medical homes
VA: veterans administration
